# Serum osteopontin is associated with coronary plaque vulnerability and short-term cardiovascular events: a prospective cohort study

**DOI:** 10.3389/fendo.2026.1771524

**Published:** 2026-02-27

**Authors:** Xingxing Wang

**Affiliations:** Department of Cardiology, Puyang Oilfield General Hospital, Puyang, Henan, China

**Keywords:** coronary artery disease, intravascular imaging, major adverse cardiovascular events, osteopontin, plaque vulnerability

## Abstract

**Background:**

Coronary plaque vulnerability underlies acute coronary events, yet reliable identification of high-risk plaques in clinical practice remains limited. Osteopontin (SPP1) is an immuno-inflammatory glycoprotein involved in atherosclerosis, but its relevance to plaque vulnerability and short-term cardiovascular events is not fully defined.

**Methods:**

In this prospective observational cohort study, a total of 300 patients were included, of whom 150 were classified as having vulnerable plaques based on IVUS/OCT imaging. Serum SPP1 and inflammatory biomarkers were measured at baseline. Plaques were classified as stable or vulnerable based on intravascular imaging. Participants were followed for 6 months to record major adverse cardiovascular events (MACE). Multivariable regression, receiver operating characteristic (ROC) analysis, survival analysis, and mediation analysis were performed to evaluate associations among SPP1, plaque vulnerability, inflammatory markers, and short-term cardiovascular events.

**Results:**

Serum SPP1 levels were significantly higher in patients with vulnerable plaques than in those with stable plaques (55.85 ± 12.26 vs. 39.18 ± 9.42 ng/mL; P < 0.001). In multivariable analyses, SPP1 was strongly associated with MMP-9 (β = 0.71) and IL-6 (β = 0.42), with a weaker association with hsCRP (β = 0.08) (all P < 0.01). In multivariable logistic regression analyses, elevated SPP1 levels were independently associated with plaque vulnerability (OR = 1.08 per unit increase; 95% CI: 1.05–1.11; P < 0.001). Receiver operating characteristic analysis demonstrated that SPP1 showed superior discriminatory performance for vulnerable plaques (AUC = 0.875, 95% CI: 0.837–0.913). During the 6-month follow-up, higher baseline SPP1 levels were independently associated with MACE (HR = 1.35; 95% CI: 1.11–1.64; P = 0.002), and Kaplan–Meier analysis showed significantly lower MACE-free survival in the high SPP1 group (log-rank P = 0.004). Mediation analysis further indicated that MMP-9 partially mediated the association between SPP1 and plaque vulnerability, accounting for 44.2% of the total effect.

**Conclusion:**

Elevated serum SPP1 levels are independently associated with imaging-defined plaque vulnerability and short-term cardiovascular events in patients with coronary artery disease. Serum SPP1 may serve as a clinically relevant biomarker reflecting immuno-inflammatory plaque instability, warranting further validation in larger cohorts.

## Introduction

Coronary artery disease (CAD) remains the leading cause of mortality worldwide (1–3). Acute coronary syndromes (ACS), the most severe clinical manifestations of CAD, are predominantly triggered by the disruption of vulnerable atherosclerotic plaques and are associated with high risks of myocardial infarction and sudden cardiac death (4, 5). Accordingly, early identification of vulnerable plaques represents a critical unmet need for preventing major adverse cardiovascular events (MACE) and improving clinical outcomes (6–8).

Despite substantial advances in cardiovascular imaging, reliable identification of plaque instability in routine clinical practice remains challenging (9, 10). Intravascular ultrasound (IVUS) and optical coherence tomography (OCT) enable detailed assessment of plaque morphology; however, their widespread application is limited by invasiveness, cost, and operator dependency (11–13). These limitations underscore the need for simple, reproducible, and minimally invasive serum biomarkers that can complement imaging techniques for individualized risk stratification and timely preventive intervention.

From a mechanistic perspective, vulnerable plaques are characterized by intense immuno-inflammatory activity, including macrophage infiltration, release of pro-inflammatory cytokines, and activation of matrix metalloproteinases (MMPs), which collectively drive extracellular matrix degradation and fibrous cap thinning (14–16). Among these mediators, MMP-9 plays a pivotal role in destabilizing the fibrous cap and is tightly regulated by upstream inflammatory signals such as interleukin-6 (IL-6) (14, 16–20). Although circulating inflammatory markers, including high-sensitivity C-reactive protein (hsCRP), have been associated with cardiovascular risk, their limited specificity and predictive performance highlight the need for biomarkers more directly linked to plaque-level inflammatory processes (18, 21, 22).

Osteopontin (SPP1) is a multifunctional immunomodulatory glycoprotein secreted by macrophages, activated T lymphocytes, and vascular smooth muscle cells (23–25). Through integrin- and CD44-mediated signaling, SPP1 promotes inflammatory cell recruitment, macrophage activation, and upregulation of MMPs, thereby contributing to extracellular matrix degradation and plaque destabilization (26–29). While experimental and cross-sectional studies have implicated SPP1 in atherosclerotic progression, its role as an upstream regulator of immuno-inflammatory plaque vulnerability and its prognostic value for short-term cardiovascular events have not been adequately validated in prospective clinical settings (23, 30).

Based on these considerations, we hypothesized that elevated serum SPP1 promotes MMP-9–mediated immuno-inflammatory responses, accelerating structural degradation of atherosclerotic plaques and increasing plaque vulnerability, thereby elevating short-term cardiovascular risk. Accordingly, this study aimed to compare serum SPP1 levels between patients with stable and vulnerable plaques, examine the associations between SPP1 and key inflammatory mediators (MMP-9, IL-6, and hsCRP), evaluate the predictive value of SPP1 for short-term MACE, and explore the potential mediating role of MMP-9 in the relationship between SPP1 and plaque vulnerability.

## Method

### Study design

This study was designed as a prospective observational cohort. Consecutive patients with angiographically confirmed coronary artery disease (CAD) were enrolled at Puyang Oilfield General Hospital between February 2023 and June 2024. At baseline, all participants underwent coronary angiography with adjunctive intravascular imaging, including intravascular ultrasound (IVUS) or optical coherence tomography (OCT), to characterize coronary plaque morphology and classify plaque vulnerability. Participants were subsequently followed for 6 months to ascertain the occurrence of major adverse cardiovascular events (MACE).

The study protocol was reviewed and approved by the Ethics Committee of Puyang Oilfield General Hospital, and written informed consent was obtained from all participants in accordance with the Declaration of Helsinki.

### Inclusion criteria and exclusion criteria

Inclusion criteria were as follows:

1. Age between 40 and 80 years;
2. Clinical diagnosis of stable angina or acute coronary syndrome (ACS) in accordance with current international guidelines;
3. Ability to provide written informed consent and willingness to comply with study procedures.

Exclusion criteria were as follows:

1. Presence of active infection or acute systemic inflammatory disease within the preceding 4 weeks;
2. Known malignant tumor or a history of cancer treatment within the past 5 years;
3. Established autoimmune or connective tissue disorders;
4. Severe hepatic dysfunction (alanine aminotransferase or aspartate aminotransferase >3 times the upper limit of normal) or severe renal dysfunction (estimated glomerular filtration rate <30 mL/min/1.73 m²);
5. Recent use of systemic immunosuppressive or corticosteroid therapy within the past 3 months;
6. Any other condition deemed by the investigators to potentially interfere with study participation or data interpretation.

### Revised grouping and follow-up

Participants were consecutively enrolled during the predefined study period according to prespecified inclusion and exclusion criteria, without consideration of plaque phenotype. After enrollment, participants were classified into two groups based on baseline intravascular imaging findings obtained by intravascular ultrasound (IVUS) and/or optical coherence tomography (OCT): a stable plaque group and a vulnerable plaque group. Plaque vulnerability was determined after enrollment using prespecified, imaging-based morphological criteria incorporating established IVUS and OCT features of plaque instability. No matching, stratified sampling, or case–control selection was applied.

Follow-up was conducted according to a predefined schedule. At baseline (T0), demographic and clinical characteristics were collected, fasting venous blood samples were obtained for biomarker measurements, and coronary plaque phenotype was assessed using IVUS and/or OCT prior to any revascularization procedures. Participants were then prospectively followed for 6 months. At the end of follow-up (T1), the occurrence of major adverse cardiovascular events (MACE) was ascertained. MACE was defined as a composite endpoint including myocardial infarction, unplanned or urgent percutaneous coronary intervention performed for recurrent ischemia or acute coronary syndromes, cardiovascular death, or rehospitalization for cardiac causes. Planned or elective revascularization procedures based on baseline intravascular imaging findings were not considered outcome events.

### Study variables and measurements

The primary exposure variable of interest was the serum concentration of osteopontin (SPP1), which was measured at baseline using a standardized enzyme-linked immunosorbent assay (ELISA). Additional circulating inflammatory biomarkers included high-sensitivity C-reactive protein (hsCRP), interleukin-6 (IL-6), and matrix metalloproteinase-9 (MMP-9). Metabolic and cardiovascular risk parameters assessed at baseline comprised low-density lipoprotein cholesterol (LDL-C), high-density lipoprotein cholesterol (HDL-C), and glycated hemoglobin (HbA1c). Renal function was evaluated using the estimated glomerular filtration rate (eGFR), calculated according to standard equations.

The clinical outcomes of interest were twofold. First, coronary plaque phenotype was classified as stable or vulnerable based on intravascular imaging findings obtained by intravascular ultrasound (IVUS) or optical coherence tomography (OCT). Second, the occurrence of major adverse cardiovascular events (MACE) during the 6-month follow-up period was recorded. MACE was defined as a composite endpoint including myocardial infarction, percutaneous coronary intervention, cardiovascular death, or rehospitalization for cardiac causes.

MACE were defined as a composite endpoint including myocardial infarction, unplanned or urgent percutaneous coronary intervention performed for recurrent ischemia or acute coronary syndromes, cardiovascular death, or rehospitalization for cardiac causes. Planned or elective revascularization procedures based on baseline intravascular imaging findings were not considered outcome events.

### Imaging assessment

Intravascular ultrasound (IVUS) was used to assess plaque burden, vessel remodeling patterns, and the presence of echolucent or echo-attenuated regions suggestive of lipid-rich plaque components. Optical coherence tomography (OCT) was applied to evaluate fibrous cap thickness, lipid-rich plaque characteristics, plaque surface morphology, and the presence of intracoronary thrombus.

Imaging-defined plaque phenotype (stable versus vulnerable) was determined using prespecified, imaging-based morphological criteria derived from established intravascular imaging literature (31, 32). For OCT, vulnerable plaque was defined by the presence of thin-cap fibroatheroma features, characterized by a lipid-rich plaque with a minimum fibrous cap thickness below established thresholds, and/or other high-risk features such as plaque rupture or intracoronary thrombus. For IVUS, surrogate markers of plaque vulnerability included large plaque burden, adverse (positive) remodeling patterns, and echolucent or attenuated regions consistent with lipid-rich plaque. When both modalities were available, OCT was prioritized for cap-based vulnerability assessment given its higher axial resolution; IVUS-based criteria were applied when OCT data were unavailable.

All intravascular imaging data were independently reviewed by two experienced cardiovascular imaging specialists who were blinded to clinical characteristics and biomarker measurements. In cases of disagreement, consensus was achieved through joint review, with adjudication by a third senior reviewer when necessary. Inter-observer reproducibility for plaque classification and key imaging measurements was assessed using appropriate agreement statistics and is reported in the **Supplementary Material**.

### Laboratory measurements

Fasting venous blood samples were collected at baseline upon hospital admission and prior to intravascular imaging and any coronary revascularization procedures, and serum was separated and stored at −80 °C until analysis. In patients presenting with acute coronary syndrome, blood sampling was performed after initial clinical stabilization but before coronary intervention, whereas in patients with stable coronary artery disease, samples were obtained under fasting conditions at admission before coronary angiography.

Serum levels of SPP1, IL-6, and MMP-9 were quantified using standardized commercial enzyme-linked immunosorbent assay (ELISA) kits according to the manufacturers’ instructions. Serum hsCRP was measured using an immunoturbidimetric assay, while other routine biochemical parameters were analyzed in the hospital’s central laboratory using automated analyzers. To ensure analytical reliability, all biomarker measurements were performed in duplicate, and results with a coefficient of variation of less than 10% were considered acceptable.

### Statistical analysis

Continuous variables were summarized as mean ± standard deviation (SD) for normally distributed data and as median with interquartile range (IQR) for non-normally distributed data. Categorical variables were expressed as frequencies and percentages. Between-group comparisons (stable versus vulnerable plaques) were performed using Student’s *t* test or the Mann–Whitney *U* test for continuous variables, as appropriate, and the chi-square test for categorical variables.

Associations between serum SPP1 levels and other inflammatory biomarkers (MMP-9, IL-6, and hsCRP) were assessed using Pearson or Spearman correlation coefficients according to data distribution. Logistic regression models were constructed to evaluate the association between serum SPP1 and imaging-defined plaque vulnerability. Receiver operating characteristic (ROC) curve analyses were performed to compare the discriminatory performance of SPP1 with other inflammatory biomarkers, with optimal cutoff values determined using the Youden index.

Covariates for multivariable logistic and Cox regression models were **pre-specified *a priori*** based on established clinical relevance to coronary plaque vulnerability and cardiovascular events, with consideration of model parsimony and the risk of overfitting. The core adjustment set included age, sex, and LDL-C for logistic regression models, and age, sex, hypertension, and LDL-C for Cox models. IL-6 was additionally included to account for systemic inflammation. Automated, data-driven variable selection procedures were not applied.

Collinearity among inflammatory biomarkers was formally assessed prior to multivariable modeling using variance inflation factors (VIFs) and tolerance statistics. A VIF value > 5 or a tolerance < 0.20 was considered indicative of potentially problematic collinearity. When SPP1 and MMP-9 were simultaneously entered into multivariable logistic regression models, both variables exceeded these thresholds, indicating substantial shared variance. Accordingly, MMP-9 was excluded from the primary multivariable logistic regression models to avoid model instability and overadjustment, while SPP1 was retained as the primary exposure variable of interest.

For longitudinal analyses, Cox proportional hazards regression models were used to assess the association between baseline serum SPP1 levels and the occurrence of major adverse cardiovascular events (MACE) during the 6-month follow-up period. Kaplan–Meier survival curves were generated to compare MACE-free survival between groups stratified by serum SPP1 levels, and differences were assessed using the log-rank test.

Mediation analyses were conducted using a nonparametric bootstrap approach with 1,000 resamples to explore the potential mediating role of MMP-9 in the association between SPP1 and plaque vulnerability. These analyses were considered exploratory and hypothesis-generating. Prespecified subgroup analyses were performed according to sex, age, diabetes, and hypertension status.

To improve clinical interpretability, serum SPP1 was additionally standardized and analyzed per standard deviation (SD) increase. Categorical analyses based on quartiles of the SPP1 distribution were also performed as sensitivity analyses. Additional sensitivity analyses included alternative modeling strategies such as log-transformation of SPP1, exclusion of outliers, and multiple imputation for missing data to assess the robustness of the findings.

All statistical analyses were performed using appropriate statistical software, and a two-sided *P* value < 0.05 was considered statistically significant.

## Results

### Baseline characteristics and follow up

Baseline characteristics of the study population are summarized in **Table 1**. A total of 300 patients were included, with 150 classified as having stable plaques and 150 as having vulnerable plaques based on IVUS/OCT findings. Patients in the vulnerable plaque group were significantly older (65.50 ± 7.15 vs. 61.34 ± 7.54 years, *P* < 0.001) and had fewer years of formal education (9.08 ± 3.22 vs. 10.18 ± 3.23 years, *P* = 0.003). Body mass index was higher in the vulnerable group (27.02 ± 3.30 vs. 25.37 ± 3.10 kg/m², *P* < 0.001), and disease duration was also longer (6.22 ± 2.56 vs. 4.70 ± 2.00 years, *P* < 0.001).

**Table 1:** Baseline characteristics of participants according to plaque type (n = 300).

Variable	Stable plaque (n = 150)	Vulnerable plaque (n = 150)	P value
Demographics			
Age (years)	61.34 ± 7.54	65.50 ± 7.15	<0.001
Sex (female), n (%)	43 (28.7%)	55 (36.7%)	0.127
Education years	10.18 ± 3.23	9.08 ± 3.22	0.003
Lifestyle and medical history			
BMI (kg/m²)	25.37 ± 3.10	27.02 ± 3.30	<0.001
Current smoking, n (%)	63 (42.0%)	56 (37.3%)	0.442
Hypertension, n (%)	77 (51.3%)	79 (52.7%)	0.885
Diabetes mellitus, n (%)	48 (32.0%)	41 (27.3%)	0.418
Disease duration (years)	4.70 ± 2.00	6.22 ± 2.56	<0.001
Laboratory parameters			
SPP1 (ng/mL)	39.18 ± 9.42	55.85 ± 12.26	<0.001
LDL-C (mmol/L)	2.80 ± 0.50	3.17 ± 0.57	<0.001
HDL-C (mmol/L)	1.11 ± 0.28	1.01 ± 0.21	0.002
eGFR (mL/min/1.73 m²)	84.45 ± 12.33	82.06 ± 14.52	0.178
hsCRP (mg/L)	2.11 ± 0.89	3.03 ± 1.19	<0.001
MMP-9 (ng/mL)	296.69 ± 53.62	395.55 ± 61.18	<0.001
IL-6 (pg/mL)	4.84 ± 1.47	6.91 ± 1.71	<0.001
HbA1c (%)	5.92 ± 0.62	6.38 ± 0.67	<0.001
Medication use			
Statin use, n (%)	80 (53.3%)	85 (56.7%)	0.634
ACEi/ARB use, n (%)	88 (58.7%)	96 (64.0%)	0.384

Values are presented as mean ± standard deviation for continuous variables and number (percentage) for categorical variables. Between-group comparisons were performed using Student’s t test or the Mann–Whitney U test for continuous variables, as appropriate, and the chi-square test for categorical variables.

BMI, body mass index; SPP1, osteopontin; LDL-C, low-density lipoprotein cholesterol; HDL-C, high-density lipoprotein cholesterol; eGFR, estimated glomerular filtration rate; hsCRP, high-sensitivity C-reactive protein; MMP-9, matrix metalloproteinase-9; IL-6, interleukin-6; HbA1c, glycated hemoglobin; ACEi, angiotensin-converting enzyme inhibitor; ARB, angiotensin receptor blocker.

Bold values represent statistically significant differences.

With respect to laboratory parameters, patients with vulnerable plaques had higher levels of LDL cholesterol (3.17 ± 0.57 vs. 2.80 ± 0.50 mmol/L, *P* < 0.001), hsCRP (3.03 ± 1.19 vs. 2.11 ± 0.89 mg/L, *P* < 0.001), MMP-9 (395.55 ± 61.18 vs. 296.69 ± 53.62 ng/mL, *P* < 0.001), IL-6 (6.91 ± 1.71 vs. 4.84 ± 1.47 pg/mL, *P* < 0.001), and HbA1c (6.38 ± 0.67 vs. 5.92 ± 0.62%, *P* < 0.001), while HDL cholesterol was significantly lower (1.01 ± 0.21 vs. 1.11 ± 0.28 mmol/L, *P* = 0.002). Estimated GFR did not differ significantly between groups. Regarding medication use, there were no significant differences in the proportions of patients receiving statins (56.7% vs. 53.3%) or ACEi/ARB therapy (64.0% vs. 58.7%). The mean SPP1 concentration in the vulnerable group was 55.85 ± 12.26 ng/mL, whereas in the stable group it was 39.18 ± 9.42 ng/mL (**Supplementary Figure 1**).

During the 6-month follow-up period, a total of 36 major adverse cardiovascular events (MACE) were documented, including 8 myocardial infarctions, 14 unplanned or clinically driven percutaneous coronary interventions, 3 cardiovascular deaths, and 11 cardiac-related rehospitalizations. A detailed breakdown of event types is provided in **Supplementary Table S1**.

### Correlation between serum SPP1 and inflammatory markers

In multivariable linear regression analyses adjusting for age, sex, and LDL-C, serum SPP1 levels were independently associated with MMP-9 (β = 0.71, 95% CI: 0.64–0.78; P < 0.001), IL-6 (β = 0.42, 95% CI: 0.34–0.50; P < 0.001), and, to a lesser extent, hsCRP (β = 0.08, 95% CI: 0.02–0.14; P = 0.009) (**Table 2**). Correlation analyses demonstrated that serum SPP1 levels were strongly and positively associated with inflammatory mediators (**Supplementary Figure 2**).

**Table 2:** Multivariable linear regression analyses of associations between inflammatory markers and serum SPP1.

Variable	β (95% CI)	P value
MMP-9	0.71 (0.64–0.78)	<0.001
IL-6	0.42 (0.34–0.50)	<0.001
hsCRP	0.08 (0.02–0.14)	0.009

Multivariable linear regression analyses were performed with serum SPP1 as the dependent variable. Models were adjusted for age, sex, and LDL-C. Standardized regression coefficients (β) with 95% confidence intervals are presented.

Bold values represent statistically significant differences.

### Logistic regression analyses for imaging-defined plaque vulnerability

In univariate logistic regression analyses, higher serum levels of SPP1, MMP-9, and IL-6 were each significantly associated with increased odds of imaging-defined plaque vulnerability (all *P* < 0.001), whereas hsCRP and LDL-C were not significantly associated with plaque vulnerability (*P* = 0.091 and *P* = 0.824, respectively) (**Table 3**).

**Table 3:** Logistic regression analyses for imaging-defined plaque vulnerability.

Variable	Univariate OR (95% CI)	P value	Multivariable OR (95% CI)*	P value
SPP1	1.19 (1.14–1.24)	<0.001	1.08 (1.05–1.11)	<0.001
MMP-9	1.16 (1.11–1.22)	<0.001	—	—
IL-6	1.49 (1.35–1.66)	<0.001	1.04 (0.99–1.09)	0.122
hsCRP	0.82 (0.65–1.03)	0.091	—	—
LDL-C	0.96 (0.65–1.41)	0.824	1.36 (1.03–1.80)	0.032
Age	—	—	1.01 (0.98–1.05)	0.416
Sex (male)	—	—	1.23 (0.69–2.18)	0.477

*The multivariable logistic regression model was pre-specified *a priori* and adjusted for age, sex, LDL-C, and IL-6. MMP-9 was not included in the multivariable model because of high collinearity with SPP1. Odds ratios are reported per unit increase in each biomarker unless otherwise specified.

In the multivariable logistic regression model adjusted for age, sex, LDL-C, and IL-6, serum SPP1 remained independently associated with plaque vulnerability (OR 1.08, 95% CI 1.05–1.11; *P* < 0.001). LDL-C also showed a modest but statistically significant association (OR 1.36, 95% CI 1.03–1.80; *P* = 0.032), whereas IL-6 was no longer statistically significant after adjustment (*P* = 0.122). Age and sex were not independently associated with plaque vulnerability in the adjusted model (both *P* > 0.05).

In expanded multivariable logistic regression models additionally adjusting for major cardiometabolic risk factors and medication use, including BMI, diabetes mellitus, current smoking status, HDL-C, HbA1c, statin use, and ACEi/ARB use, the association between serum SPP1 and imaging-defined plaque vulnerability remained robust and statistically significant (**Supplementary Table S1**).

### Cox regression analysis for prediction of MACE

Cox proportional hazards models were used to evaluate the prognostic value of serum biomarkers for 6-month MACE (**Table 4**). In univariate analyses, higher levels of SPP1, MMP-9, and IL-6 were significantly associated with increased risk of MACE. Specifically, SPP1 showed the strongest association (HR 1.47, 95% CI: 1.23–1.76, *P* < 0.001), followed by MMP-9 (HR 1.31, 95% CI: 1.11–1.55, *P* = 0.001) and IL-6 (HR 1.25, 95% CI: 1.05–1.50, *P* = 0.011). hs-CRP was not predictive of MACE (*P* = 0.262). In the multivariate model adjusting for age, sex, and hypertension, SPP1 remained an independent predictor of 6-month MACE (HR 1.35, 95% CI: 1.11–1.64, *P* = 0.002). In contrast, the associations of MMP-9 and IL-6 with MACE were attenuated and no longer statistically significant (both *P*>0.05).

**Table 4:** Cox proportional hazards regression analyses for prediction of 6-month MACE.

Variable	Univariate HR (95% CI)	P value	Multivariable HR (95% CI)*	P value
SPP1	1.47 (1.23–1.76)	<0.001	1.35 (1.11–1.64)	0.002
MMP-9	1.31 (1.11–1.55)	0.001	1.18 (0.98–1.43)	0.082
IL-6	1.25 (1.05–1.50)	0.011	1.09 (0.90–1.33)	0.385
hsCRP	1.12 (0.92–1.36)	0.262	1.06 (0.84–1.33)	0.642
Age	—	—	1.02 (0.99–1.05)	0.196
Sex (male)	—	—	1.21 (0.68–2.15)	0.509
Hypertension	—	—	1.52 (0.89–2.60)	0.124

*Multivariable Cox model adjusted for age, sex, hypertension, IL-6, and LDL-C. MMP-9 was included but did not remain statistically significant after adjustment. Hazard ratios are reported per unit increase in serum biomarker levels.

In expanded multivariable Cox proportional hazards models additionally adjusting for major cardiometabolic risk factors and medication use, including BMI, diabetes mellitus, current smoking status, HDL-C, HbA1c, and statin use, baseline serum SPP1 remained independently associated with an increased risk of 6-month major adverse cardiovascular events (**Supplementary Table S2**).

### Associations of serum SPP1 expressed per standard deviation and by quartiles

When serum SPP1 was re-parameterized to improve clinical interpretability, analyses based on standard deviation (SD) increase and distribution-based quartiles yielded results consistent with the primary per-unit models. In quartile-based analyses, compared with the lowest SPP1 quartile, participants in the highest quartile exhibited markedly increased risks of plaque vulnerability (OR = 2.74, 95% CI 1.78–4.23, *P* < 0.001) and 6-month MACE (HR = 2.36, 95% CI 1.38–4.04, *P* = 0.002), with a clear graded increase in effect estimates across higher quartiles. These findings indicate a dose–response relationship between serum SPP1 levels and both plaque instability and short-term cardiovascular risk (**Supplementary Table S3**).

### Sensitivity analyses adjusting for renal function

To address potential confounding by renal function, sensitivity analyses were performed with additional adjustment for baseline estimated glomerular filtration rate (eGFR). In logistic regression models evaluating imaging-defined plaque vulnerability, the association between serum SPP1 levels and plaque vulnerability remained statistically significant after inclusion of eGFR in the multivariable model (OR per unit increase, 1.07; 95% CI, 1.04–1.10; *P* < 0.001), with minimal attenuation compared with the primary model. Baseline eGFR itself was not independently associated with plaque vulnerability (*P* = 0.318).

Similarly, in Cox proportional hazards models for 6-month major adverse cardiovascular events, additional adjustment for eGFR did not materially alter the association between SPP1 and incident MACE (HR per unit increase, 1.33; 95% CI, 1.09–1.62; *P* = 0.004). Renal function was not a significant predictor of MACE in the fully adjusted model (*P* = 0.176). (**Supplementary Table S4**).

### ROC curve analysis for predicting plaque rupture

**Figure 1:**
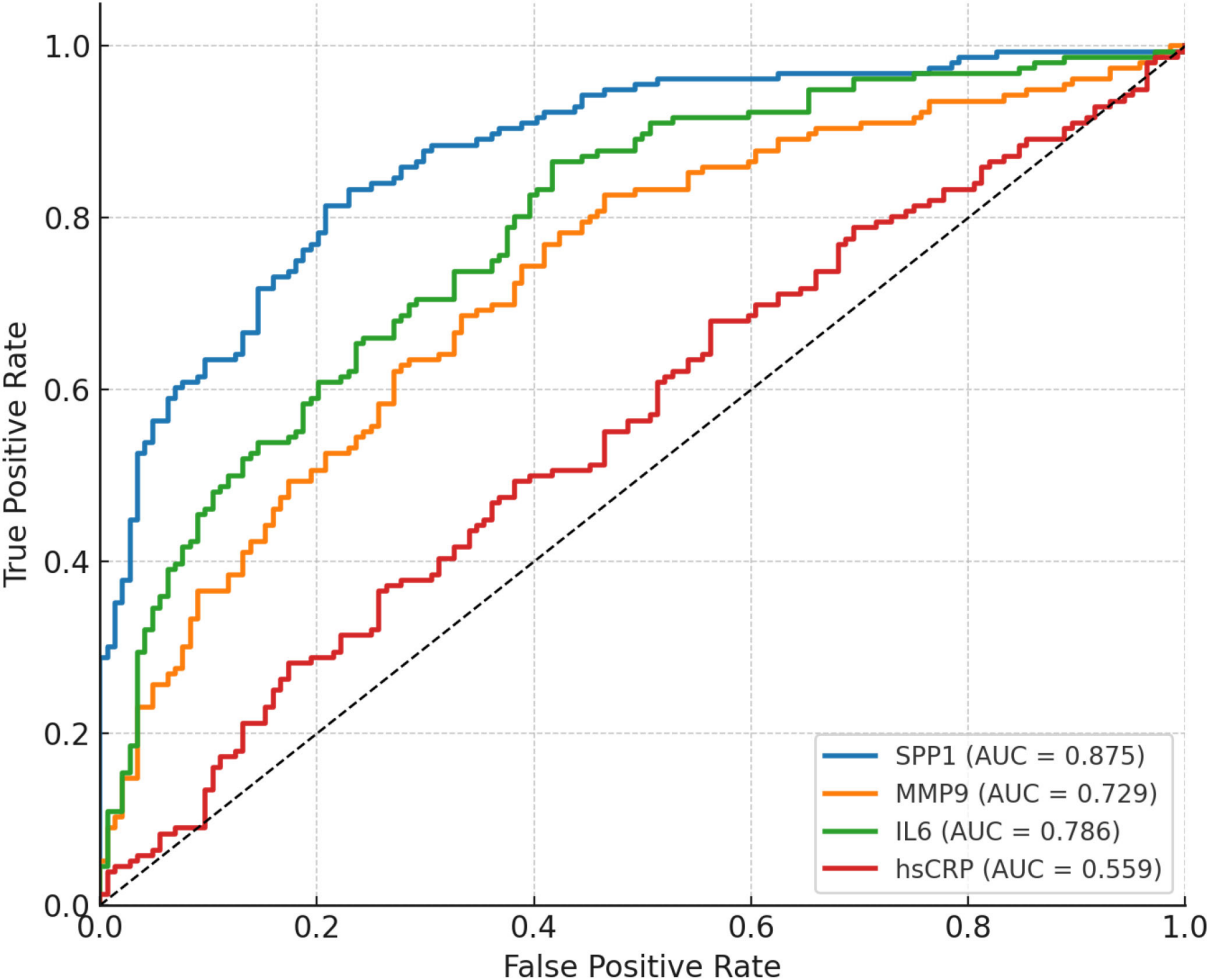
ROC curves for serum biomarkers predicting plaque rupture.

Receiver operating characteristic (ROC) curve analysis was performed to compare the diagnostic performance of serum biomarkers in discriminating vulnerable from stable plaques (**Figure 1**; **Table 5**). Among all tested markers, SPP1 demonstrated the highest diagnostic accuracy, with an area under the curve (AUC) of 0.875 (95% CI: 0.837–0.913). In comparison, IL-6 (AUC = 0.786, 95% CI: 0.736–0.836) and MMP-9 (AUC = 0.729, 95% CI: 0.673–0.786), while hs-CRP demonstrated poor discrimination (AUC = 0.559, 95% CI: 0.491–0.625). Pairwise comparisons of ROC curves using DeLong’s test demonstrated that the AUC of SPP1 was significantly higher than those of IL-6, MMP-9, and hsCRP (**Supplementary Table S5**).

**Table 5:** Diagnostic performance of biomarkers in predicting plaque rupture.

Biomarker	AUC	95% CI	Sensitivity	Specificity	Youden index
SPP1	0.875	(0.837, 0.913)	0.81	0.79	0.61
IL-6	0.786	(0.736, 0.836)	0.87	0.58	0.45
MMP-9	0.729	(0.673, 0.786)	0.83	0.53	0.36
hs-CRP	0.559	(0.491, 0.625)	0.68	0.44	0.12

### Kaplan–Meier survival analysis

**Figure 2:**
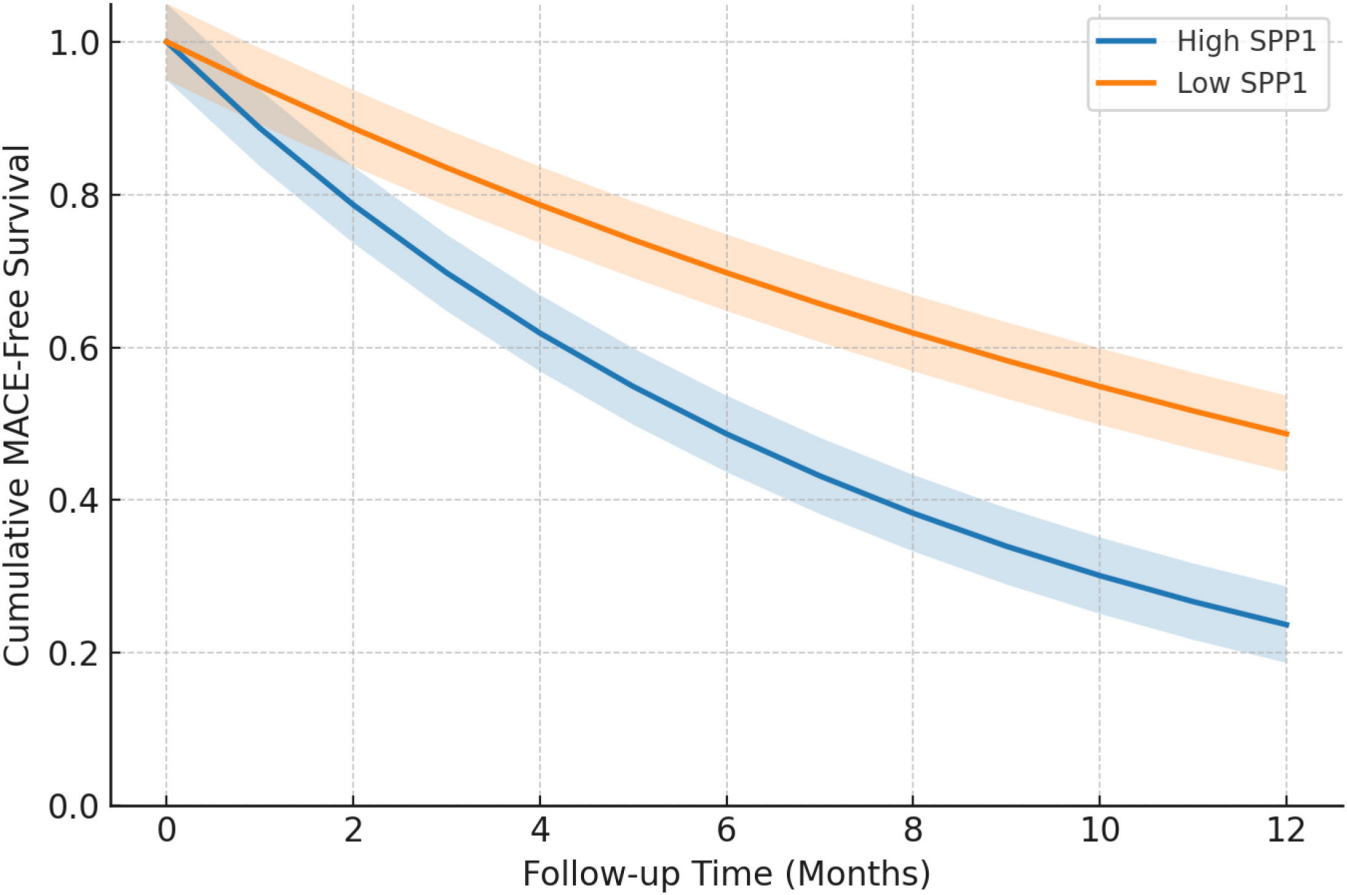
Kaplan–Meier curves for cumulative MACE-free survival stratified by serum SPP1 levels.

Kaplan–Meier survival curves demonstrated a significant difference in cumulative MACE-free survival between patients stratified by serum SPP1 levels (**Figure 2**). Patients were dichotomized into high and low SPP1 groups based on the median serum SPP1 concentration of the study population. For Kaplan–Meier analyses, patients were stratified into high and low SPP1 groups based on the median serum SPP1 concentration of the overall study population (median value: 47.0 ng/mL). During the 6-month follow-up period, patients in the high SPP1 group experienced a higher incidence of MACE compared with those in the low SPP1 group. Separation of event-free survival curves was observed early during follow-up and became more pronounced over time, with MACE-free survival declining to below 70% in the high SPP1 group by the end of follow-up. The difference between groups was statistically significant according to the log-rank test (*P* = 0.004).

### Mediation analysis

To further explore the mechanistic pathway linking SPP1 to plaque rupture, a mediation analysis was conducted using the bootstrap method (**Table 6**). The total effect of SPP1 on plaque rupture was significant (estimate = 0.423, 95% CI: 0.271–0.576, *P* < 0.001). Of this, an indirect effect mediated through MMP-9 accounted for 0.187 (95% CI: 0.072–0.318, *P* < 0.01), representing 44.2% of the total effect. The direct effect of SPP1 on plaque rupture remained significant (estimate = 0.236, 95% CI: 0.065–0.407).

**Table 6:** Mediation analysis evaluating MMP-9 as a mediator in the association between SPP1 and imaging-defined plaque vulnerability.

Effect type	Estimate	95% CI	P value
Indirect effect (SPP1 → MMP-9 → plaque vulnerability)	0.187	0.072–0.318	<0.01
Direct effect (SPP1 → plaque vulnerability)	0.236	0.065–0.407	<0.01
Total effect	0.423	0.271–0.576	<0.001
Proportion mediated	44.2%	—	—

Mediation analysis was performed using a nonparametric bootstrap approach with 1,000 resamples. Estimates represent standardized regression coefficients derived from logistic regression–based mediation models. Indirect, direct, and total effects were considered statistically significant when the 95% confidence interval did not include zero.

### Subgroup analyses and sensitivity analyses

The association of higher serum SPP1 with plaque rupture and 6-month MACE was consistent across sex, age, diabetes, hypertension, LDL, statin use, smoking, and BMI subgroups. Effect sizes were comparable (ORs 1.55–1.68; HRs 1.38–1.49) with no significant interactions (all P-interaction >0.3) (**Supplementary Tables S6, S7**). Results remained robust when SPP1 was modeled as log-transformed, dichotomized at the ROC cutoff or median, after trimming outliers or imputing missing data. Adjustment for MMP-9 and IL-6 attenuated but did not abolish the association (OR 1.38, P = 0.006) (**Supplementary Table S8**). SPP1 consistently predicted 6-month MACE under multiple specifications, including log-transformation, dichotomization, statin-restricted models, IPTW, and multiple imputation. Alternative endpoint definitions yielded similar results, and proportional hazards assumptions were satisfied (**Supplementary Table S9**).

## Discussion

In this prospective cohort study of patients with angiographically confirmed coronary artery disease who underwent IVUS/OCT for plaque characterization, we systematically evaluated the clinical relevance of serum SPP1 in relation to imaging-defined plaque vulnerability and short-term cardiovascular events. To our knowledge, this is the first study to comprehensively examine serum SPP1 across imaging, inflammatory biomarker, and short-term clinical outcome dimensions within a single prospective framework. We found that serum SPP1 levels were significantly higher in patients with vulnerable plaques and were strongly correlated with key inflammatory mediators, particularly MMP-9 and IL-6. Multivariable logistic regression analyses demonstrated that SPP1 was independently associated with plaque vulnerability, and ROC analyses showed superior discriminatory performance compared with traditional inflammatory markers. Furthermore, elevated SPP1 levels were consistently associated with a higher incidence of 6-month major adverse cardiovascular events in Cox regression and Kaplan–Meier analyses. Mediation analyses further suggested that MMP-9 partially mediated the association between SPP1 and plaque vulnerability. Collectively, these findings support serum SPP1 as a clinically relevant biomarker reflecting immuno-inflammatory plaque vulnerability and its association with subsequent short-term cardiovascular events.

CAD is a progressive condition in which adverse clinical outcomes are predominantly driven by acute plaque destabilization rather than the extent of luminal stenosis alone (33, 34). MACE remain common despite advances in pharmacological therapy and revascularization strategies, highlighting the limitations of conventional risk stratification approaches (35–37). In this context, identifying biomarkers that reflect the underlying biological processes linking coronary plaque vulnerability to subsequent clinical events is of substantial clinical relevance. Our findings support the concept that immuno-inflammatory activity within atherosclerotic plaques contributes to short-term cardiovascular events and may partially explain the residual risk observed in patients with established CAD.

Previous studies have implicated SPP1 in multiple aspects of atherosclerosis, including vascular inflammation, macrophage activation, and extracellular matrix remodeling (38, 39). Experimental and pathological evidence has demonstrated increased SPP1 expression within atherosclerotic lesions, particularly in regions enriched with inflammatory infiltrates and matrix degradation, supporting its involvement in plaque progression and destabilization (40, 41). Extending these observations, the present study demonstrates that elevated serum SPP1 is closely associated with imaging-defined plaque vulnerability and is independently associated with subsequent short-term cardiovascular events. These findings suggest that circulating SPP1 reflects plaque-level immuno-inflammatory activity relevant to clinical instability.

Compared with prior studies, several features strengthen the current evidence. The prospective cohort design reduces retrospective bias and enables temporal assessment between biomarker levels and clinical outcomes. In addition, the integration of high-resolution intravascular imaging (IVUS/OCT) with circulating biomarker measurements allows for more accurate characterization of plaque phenotypes and their biological correlates. Finally, the inclusion of longitudinal follow-up with survival analyses provides clinical context, supporting the relevance of SPP1 beyond cross-sectional associations. Collectively, these aspects highlight the incremental value of the present study in clarifying the clinical and biological significance of SPP1 in coronary artery disease.

SPP1 is increasingly recognized as a multifunctional cytokine-like glycoprotein that modulates immuno-inflammatory activity within atherosclerotic lesions (42, 43). It promotes the recruitment and migration of inflammatory cells, particularly macrophages and activated T lymphocytes, and enhances the expression of matrix metalloproteinases, with MMP-9 serving as a key effector involved in extracellular matrix remodeling (26, 44, 45). Through integrin- and CD44-mediated signaling pathways, SPP1 contributes to smooth muscle cell phenotypic modulation, extracellular matrix degradation, and adverse vascular remodeling, processes that collectively favor fibrous cap thinning and plaque destabilization (27, 46, 47).

Previous studies have consistently demonstrated that imaging-defined vulnerable coronary plaques are strongly associated with subsequent adverse cardiovascular outcomes (48, 49). Large-scale prospective investigations using intravascular imaging have shown that high-risk plaque features, such as thin-cap fibroatheroma and adverse remodeling, confer a significantly increased risk of future myocardial infarction and other major adverse cardiovascular events, even after adjustment for traditional risk factors (48, 49). In this context, the present study extends prior evidence by linking plaque vulnerability not only to clinical outcomes but also to a circulating inflammatory biomarker profile. By integrating serum SPP1 levels with intravascular imaging findings, our results provide additional insight into the inflammatory milieu underlying plaque instability and suggest a potential role for biomarker-informed risk stratification in patients with coronary artery disease.

Previous studies have explored the relationship between circulating biomarkers and imaging-defined vulnerable coronary plaques (50, 51). Prior investigations using intravascular imaging have reported associations between inflammatory and extracellular matrix–related biomarkers and high-risk plaque features, including lipid-rich plaques, thin fibrous caps, and adverse remodeling patterns (50, 51). These studies suggest that systemic inflammatory activity is reflected in local plaque morphology and vulnerability, although the reported biomarkers and imaging criteria have varied across cohorts. Collectively, this body of evidence supports a link between circulating biomarker profiles and plaque instability, providing a biological context for the present findings.

In the present study, the strong independent association observed between serum SPP1 and MMP-9 provides clinical support for a coordinated immuno-inflammatory and matrix-degrading pathway underlying plaque vulnerability. Mediation analysis further suggested that MMP-9 partially mediated the association between SPP1 and imaging-defined plaque vulnerability, accounting for a substantial proportion of the total effect. Together, these findings indicate that SPP1 may reflect upstream immuno-inflammatory activity linked to matrix degradation and structural weakening of atherosclerotic plaques. This integrative role supports the relevance of SPP1 as a biomarker of plaque vulnerability and its association with subsequent short-term cardiovascular events.

From a biomarker perspective, serum SPP1 may represent a complementary indicator for identifying plaque vulnerability beyond conventional clinical and inflammatory markers. The incorporation of SPP1 into clinical assessment could potentially improve individualized risk stratification by reflecting underlying immuno-inflammatory plaque activity. In particular, the combined evaluation of baseline serum SPP1 levels with IVUS/OCT imaging may provide a more integrated framework for characterizing plaque phenotype and short-term clinical risk. Patients with elevated SPP1 levels may therefore warrant closer clinical follow-up and optimized preventive management, although further studies are needed to determine how SPP1-guided strategies could be incorporated into routine practice.

An important observation in the present study is that the associations of IL-6 and MMP-9 with plaque vulnerability and short-term cardiovascular events were attenuated and no longer statistically significant after multivariable adjustment, as reflected by confidence intervals crossing the null value. This finding should be interpreted in the context of overlapping inflammatory pathways rather than as evidence against the biological relevance of these markers. IL-6 and MMP-9 are well-established downstream mediators of vascular inflammation and matrix remodeling; however, their circulating levels may partly reflect common upstream inflammatory activity captured by SPP1. Consistent with this interpretation, SPP1 showed strong correlations with both IL-6 and MMP-9 and remained independently associated with imaging-defined plaque vulnerability and 6-month MACE after adjustment. Thus, the attenuation of IL-6 and MMP-9 in multivariable models likely reflects shared variance and potential mediation rather than the absence of an underlying inflammatory contribution.

The relatively short follow-up duration represents an important consideration in interpreting the prognostic findings of the present study. This investigation was specifically designed to assess the short-term clinical implications of imaging-defined plaque vulnerability in relation to circulating SPP1 levels. A 6-month follow-up period was selected *a priori* to capture early adverse cardiovascular events, which are more likely to be temporally linked to plaque instability identified at baseline intravascular imaging. Although longer follow-up would be required to evaluate long-term cardiovascular risk, prior intravascular imaging studies have demonstrated that vulnerable plaque features are particularly predictive of early clinical events. In this context, the present findings support the potential value of SPP1 as a biomarker associated with short-term risk stratification. Nonetheless, the limited follow-up duration precludes conclusions regarding long-term outcomes, which should be addressed in future studies with extended follow-up.

This study has several limitations. First, it was conducted at a single center with a moderate sample size, which may limit the generalizability of the findings. Second, the number of major adverse cardiovascular events was relatively small and the follow-up period was short, precluding assessment of long-term clinical outcomes. Third, plaque classification was based on expert interpretation of IVUS/OCT imaging; although consensus adjudication was applied, a degree of subjectivity cannot be completely excluded. Although intravascular imaging was used at baseline, planned revascularization procedures were excluded from the MACE definition to minimize potential bias related to outcome ascertainment. In addition, the mediation analysis reflects statistical associations rather than causal relationships and should be interpreted as exploratory.

Future studies should aim to validate these findings in larger, multicenter cohorts with longer follow-up to clarify the longitudinal relationship between serum SPP1 levels, plaque vulnerability, and cardiovascular events. Further investigations are also warranted to examine the role of SPP1 across different plaque phenotypes and to explore whether combining SPP1 with other biomarkers could improve risk stratification. Finally, experimental studies are needed to elucidate the biological mechanisms underlying the SPP1–MMP-9 pathway and its contribution to plaque destabilization.

## Conclusion

In this prospective cohort study, elevated serum SPP1 levels were independently associated with imaging-defined plaque vulnerability and short-term major adverse cardiovascular events in patients with coronary artery disease. SPP1 showed better discriminatory performance than traditional inflammatory markers, and mediation analyses suggested partial involvement of MMP-9 in this association. These findings support serum SPP1 as a clinically relevant biomarker reflecting plaque vulnerability and short-term cardiovascular events, warranting further validation in larger studies.

## Data Availability

The original contributions presented in the study are included in the article/**Supplementary Material**. Further inquiries can be directed to the corresponding author.
