# Supplementary material for: Serum osteopontin is associated with coronary plaque vulnerability and short-term cardiovascular events: a prospective cohort study
Source: Front Endocrinol (Lausanne). 2026 Feb 27;17:1771524. doi: 10.3389/fendo.2026.1771524 (PMC12982015; doi:10.3389/fendo.2026.1771524)
Supplement: Supplementary file 1 [file DataSheet1.pdf]

Python Analysis Script Template (Exported as PDF)

Study: Serum SPP1, coronary plaque vulnerability (IVUS/OCT), and 6-month MACE.

Generated: 2025-12-19

How to use this template

This document provides a ready-to-adapt Python workflow aligned to the analyses described in your manuscript: baseline comparisons (Table 1), multivariable linear regression (Table 2), logistic regression (Table 3), ROC (Table 4), Cox + Kaplan-Meier (Table 5 / Figure 2), mediation (Table 6), and subgroup/sensitivity analyses (Supplement). Replace the placeholder file path and column names to match your dataset.

Full script

```
# ============================================================================
# Python Analysis Script Template
# Study: Serum osteopontin (SPP1) and coronary plaque vulnerability + 6-month MACE
# Design: Prospective observational cohort with IVUS/OCT plaque classification
# ============================================================================

# 0) Environment
# ----------------------------------------------------------------------------
# Recommended Python >= 3.10
# pip install pandas numpy scipy statsmodels scikit-learn lifelines matplotlib pingouin

import numpy as np
import pandas as pd

from scipy import stats
import statsmodels.api as sm
import statsmodels.formula.api as smf

from sklearn.metrics import roc_auc_score, roc_curve

from lifelines import CoxPHFitter, KaplanMeierFitter
from lifelines.statistics import logrank_test

import matplotlib.pyplot as plt

# 1) Load data
# ----------------------------------------------------------------------------
# Expected wide-format dataset (one row per participant).
# REQUIRED COLUMNS (rename as needed):
#   id, plaque (0=stable, 1=vulnerable), time_months, mace (0/1)
#   spp1, mmp9, il6, hscrp
#   age, sex (0=female, 1=male), ldl_c, hdl_c, bmi, hba1c, egfr
#   hypertension, diabetes, smoking, statin, acei_arb
#
# NOTE: If follow-up time is fixed at 6 months for all participants, set time_months = 6.

df = pd.read_csv("data.csv")  # <-- replace

# Basic checks
assert df["plaque"].isin([0, 1]).all()
assert df["mace"].isin([0, 1]).all()

# 2) Descriptive statistics and group comparisons (Table 1)
```

```
# ----------------------------------------------------------------------------
def compare_cont(x_stable, x_vuln):
    # Use t-test if both groups are approximately normal; otherwise Mann-Whitney U.
    p1 = stats.shapiro(x_stable.dropna()).pvalue if len(x_stable.dropna()) < 5000 else 0.0
    p2 = stats.shapiro(x_vuln.dropna()).pvalue if len(x_vuln.dropna()) < 5000 else 0.0
    if (p1 > 0.05) and (p2 > 0.05):
        _, p = stats.ttest_ind(x_stable, x_vuln, nan_policy="omit", equal_var=False)
    else:
        _, p = stats.mannwhitneyu(x_stable.dropna(), x_vuln.dropna(), alternative="two-sided")
    return p

stable = df[df["plaque"] == 0]
vuln   = df[df["plaque"] == 1]

# Example: SPP1 P value
p_spp1 = compare_cont(stable["spp1"], vuln["spp1"])
print("SPP1 group difference P:", p_spp1)

# 3) Multivariable linear regression (Table 2) - standardized betas
# ----------------------------------------------------------------------------
# Model: spp1 ~ mmp9 + il6 + hscrp + age + sex + ldl_c
cols_z = ["spp1", "mmp9", "il6", "hscrp", "age", "ldl_c"]
df_z = df.copy()
for c in cols_z:
    df_z[c] = (df_z[c] - df_z[c].mean()) / df_z[c].std(ddof=0)

ols = smf.ols("spp1 ~ mmp9 + il6 + hscrp + age + sex + ldl_c", data=df_z).fit(cov_type="HC3")
print(ols.summary())

# 4) Logistic regression for plaque vulnerability (Table 3)
# ----------------------------------------------------------------------------
logit = smf.logit("plaque ~ spp1 + age + sex + ldl_c", data=df).fit(disp=False)
or_ = np.exp(logit.params)
ci = np.exp(logit.conf_int())
print(pd.DataFrame({"OR": or_, "CI_low": ci[0], "CI_high": ci[1], "p": logit.pvalues}))

# 5) ROC analysis (Table 4 / Figure 1)
# ----------------------------------------------------------------------------
y = df["plaque"].values
auc_spp1 = roc_auc_score(y, df["spp1"].values)
print("AUC (SPP1):", auc_spp1)

fpr, tpr, _ = roc_curve(y, df["spp1"].values)
plt.figure()
plt.plot(fpr, tpr)
plt.plot([0, 1], [0, 1], linestyle="--")
plt.xlabel("False positive rate")
plt.ylabel("True positive rate")
plt.title("ROC - SPP1 for vulnerable plaque")
plt.tight_layout()
plt.savefig("roc_spp1.png", dpi=300)

# 6) Cox regression for MACE during follow-up (Table 5)
# ----------------------------------------------------------------------------
cox_df = df[["time_months", "mace", "spp1", "age", "sex", "hypertension"]].dropna().copy()
cph = CoxPHFitter()
cph.fit(cox_df, duration_col="time_months", event_col="mace")
cph.print_summary()

# 7) Kaplan-Meier curves + log-rank test (Figure 2)
# ----------------------------------------------------------------------------
cut = df["spp1"].median()
df["spp1_high"] = (df["spp1"] >= cut).astype(int)
```

```
kmf = KaplanMeierFitter()
plt.figure()
for grp, lab in [(0, "Low SPP1"), (1, "High SPP1")]:
    mask = df["spp1_high"] == grp
    kmf.fit(df.loc[mask, "time_months"], df.loc[mask, "mace"], label=lab)
    kmf.plot_survival_function()
plt.xlabel("Months")
plt.ylabel("MACE-free survival")
plt.title("Kaplan-Meier - MACE by SPP1")
plt.tight_layout()
plt.savefig("km_spp1.png", dpi=300)

low = df[df["spp1_high"] == 0]
high = df[df["spp1_high"] == 1]
lr = logrank_test(low["time_months"], high["time_months"], event_observed_A=low["mace"], event_observed_[illegible]
print("Log-rank P:", lr.p_value)

# 8) Mediation analysis (Table 6) - bootstrap (exploratory)
# ---------------------------------------------------------------------------
# X=SPP1, M=MMP-9, Y=plaque (binary). Interpret on the log-odds scale.
import numpy.random as npr

def mediation_boot(df_in, n_boot=1000, seed=123):
    rng = npr.default_rng(seed)
    ind_list, dir_list, tot_list = [], [], []
    cov = "age + sex + ldl_c"
    for _ in range(n_boot):
        d = df_in.sample(frac=1, replace=True, random_state=int(rng.integers(0, 1e9)))
        a = smf.ols(f"mmp9 ~ spp1 + {cov}", data=d).fit().params["spp1"]
        b_fit = smf.logit(f"plaque ~ spp1 + mmp9 + {cov}", data=d).fit(disp=False)
        b = b_fit.params["mmp9"]
        c_prime = b_fit.params["spp1"]
        c_tot = smf.logit(f"plaque ~ spp1 + {cov}", data=d).fit(disp=False).params["spp1"]
        ind_list.append(a * b)
        dir_list.append(c_prime)
        tot_list.append(c_tot)
    return np.array(ind_list), np.array(dir_list), np.array(tot_list)

df_med = df[["plaque", "spp1", "mmp9", "age", "sex", "ldl_c"]].dropna()
ind, direct, total = mediation_boot(df_med, n_boot=1000)

def ci95(x):
    return np.percentile(x, 2.5), np.percentile(x, 97.5)

print("Indirect (a*b):", np.mean(ind), ci95(ind))
print("Direct (c'):", np.mean(direct), ci95(direct))
print("Total (c):", np.mean(total), ci95(total))
print("Proportion mediated:", np.mean(ind) / np.mean(total))
```
