# Supplementary material for: Serum osteopontin is associated with coronary plaque vulnerability and short-term cardiovascular events: a prospective cohort study
Source: Front Endocrinol (Lausanne). 2026 Feb 27;17:1771524. doi: 10.3389/fendo.2026.1771524 (PMC12982015; doi:10.3389/fendo.2026.1771524)
Supplement: Supplementary file 3 [file DataSheet3.docx]

**Supplementary** Material

**Inter-observer reproducibility for intravascular imaging measurements and plaque classification**

**A. Inter-observer agreement for categorical plaque classification**

| **Classification outcome** | **Number of lesions** | **Cohen’s κ (95% CI)** | **P value** |
| --- | --- | --- | --- |
| Plaque phenotype (stable vs vulnerable) | 162 | 0.82 (0.75–0.88) | <0.001 |

**B. Inter-observer reproducibility for continuous intravascular imaging measurements**

| **Imaging parameter** | **Imaging modality** | **Number of lesions** | **ICC (95% CI)** | **P value** |
| --- | --- | --- | --- | --- |
| Fibrous cap thickness | OCT | 98 | 0.88 (0.83–0.92) | <0.001 |
| Plaque burden | IVUS | 162 | 0.85 (0.79–0.90) | <0.001 |
| Lipid-rich plaque extent | OCT | 98 | 0.81 (0.74–0.87) | <0.001 |

Inter-observer agreement for categorical plaque classification was assessed using Cohen’s kappa (κ). Inter-observer reproducibility for continuous imaging measurements was evaluated using intraclass correlation coefficients (ICC) based on a two-way random-effects model with absolute agreement.

All imaging analyses were independently performed by two experienced cardiovascular imaging specialists blinded to clinical and biomarker data. Discrepancies were resolved by consensus, with adjudication by a third senior reviewer when required.

P values are two-sided, and values < 0.05 indicate statistically significant agreement beyond chance.

‘

**
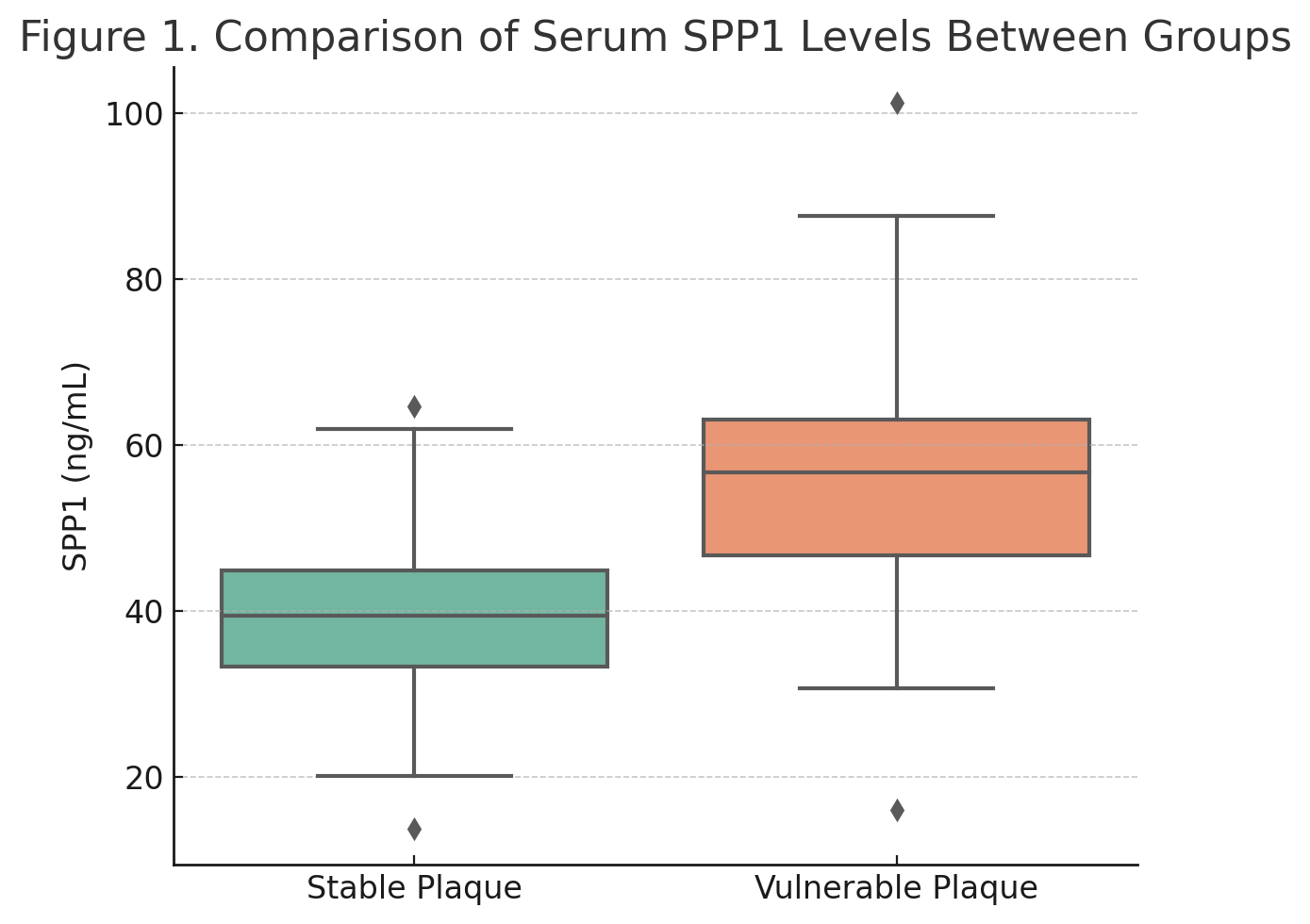
**

**Supplementary Figure 1. Comparison of serum SPP1 levels between stable and vulnerable plaque groups. A significant elevation of SPP1 was observed in the vulnerable group (P < 0.001).**

**Supplementary Table S1. Composition of major adverse cardiovascular events during 6-month follow-up (n = 300)**

| **Event type** | **Number of events, n** |
| --- | --- |
| Myocardial infarction | 8 |
| Percutaneous coronary intervention | 14 |
| Cardiovascular death | 3 |
| Cardiac-related rehospitalization | 11 |
| **Total MACE** | **36** |

**Table footnotes**

Major adverse cardiovascular events (MACE) were defined as a composite endpoint including myocardial infarction, percutaneous coronary intervention, cardiovascular death, or rehospitalization for cardiac causes during the 6-month follow-up period. Event counts are presented as the number of first events contributing to the composite endpoint.

**
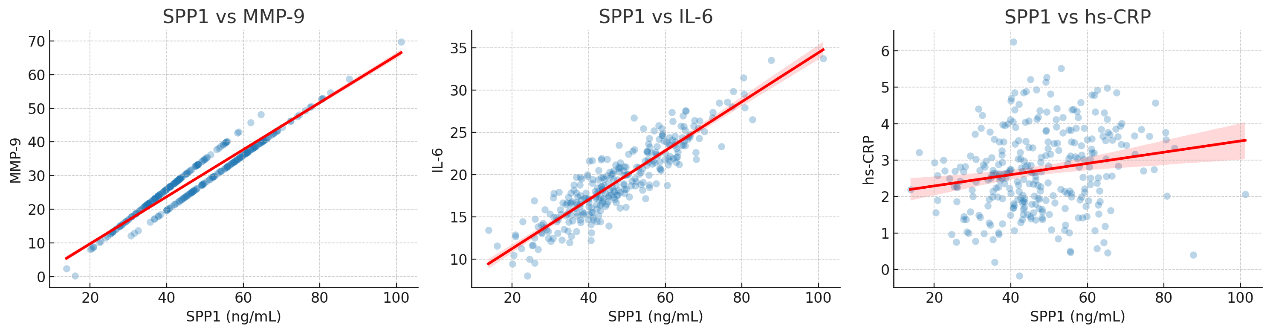
**

**Supplementary Figure 2. Correlation between serum SPP1 levels and inflammatory markers.**

**Supplementary Table S1. Expanded multivariable logistic regression models for imaging-defined plaque vulnerability**

| **Variable** | **Model 1 OR (95% CI)** | **P value** | **Model 2 OR (95% CI)** | **P value** |
| --- | --- | --- | --- | --- |
| **SPP1** | 1.08 (1.05–1.11) | <0.001 | 1.07 (1.04–1.10) | <0.001 |
| Age | 1.01 (0.98–1.05) | 0.416 | 1.01 (0.97–1.04) | 0.528 |
| Sex (male) | 1.23 (0.69–2.18) | 0.477 | 1.18 (0.66–2.11) | 0.584 |
| LDL-C | 1.36 (1.03–1.80) | 0.032 | 1.29 (0.98–1.70) | 0.067 |
| IL-6 | 1.04 (0.99–1.09) | 0.122 | 1.03 (0.98–1.08) | 0.214 |
| BMI | — | — | 1.05 (0.99–1.11) | 0.083 |
| Diabetes mellitus | — | — | 1.21 (0.74–1.98) | 0.446 |
| Current smoking | — | — | 1.12 (0.68–1.84) | 0.653 |
| HDL-C | — | — | 0.79 (0.52–1.19) | 0.258 |
| HbA1c | — | — | 1.14 (0.88–1.48) | 0.329 |
| Statin use | — | — | 0.91 (0.55–1.51) | 0.721 |
| ACEi/ARB use | — | — | 0.95 (0.58–1.56) | 0.841 |

**Table footnote:**
Model 1 represents the primary *a priori* multivariable logistic regression model adjusted for age, sex, LDL-C, and IL-6.
Model 2 represents the expanded model, additionally adjusting for BMI, diabetes mellitus, current smoking status, HDL-C, HbA1c, statin use, and ACEi/ARB use.
Odds ratios (ORs) are reported per unit increase in continuous variables. P values are two-sided, with statistical significance defined as *P* < 0.05.

**Supplementary Table S2. Expanded multivariable Cox regression models for 6-month major adverse cardiovascular events (MACE)**

| **Variable** | **Model 1 HR (95% CI)** | **P value** | **Model 2 HR (95% CI)** | **P value** |
| --- | --- | --- | --- | --- |
| **SPP1** | 1.35 (1.11–1.64) | 0.002 | 1.33 (1.09–1.61) | 0.004 |
| Age | 1.02 (0.99–1.05) | 0.196 | 1.02 (0.99–1.05) | 0.214 |
| Sex (male) | 1.21 (0.68–2.15) | 0.509 | 1.17 (0.65–2.09) | 0.598 |
| Hypertension | 1.52 (0.89–2.60) | 0.124 | 1.44 (0.83–2.49) | 0.193 |
| LDL-C | 1.18 (0.92–1.52) | 0.191 | 1.14 (0.88–1.47) | 0.321 |
| IL-6 | 1.09 (0.90–1.33) | 0.385 | 1.07 (0.88–1.31) | 0.487 |
| BMI | — | — | 1.03 (0.97–1.09) | 0.327 |
| Diabetes mellitus | — | — | 1.28 (0.77–2.12) | 0.339 |
| Current smoking | — | — | 1.19 (0.71–2.00) | 0.505 |
| HDL-C | — | — | 0.82 (0.55–1.22) | 0.323 |
| HbA1c | — | — | 1.17 (0.90–1.52) | 0.242 |
| Statin use | — | — | 0.88 (0.53–1.46) | 0.615 |

**Table footnote:**
Model 1 represents the primary *a priori* Cox proportional hazards model adjusted for age, sex, hypertension, LDL-C, and IL-6.
Model 2 represents the expanded model, additionally adjusting for BMI, diabetes mellitus, current smoking status, HDL-C, HbA1c, and statin use.
Hazard ratios (HRs) are reported per unit increase in continuous variables. P values are two-sided, with statistical significance defined as *P* < 0.05.

**Supplementary Table S3. Associations of serum SPP1 with plaque vulnerability and 6-month major adverse cardiovascular events expressed per standard deviation increase and by quartiles**

**A. Logistic regression analyses for imaging-defined plaque vulnerability**

| **Exposure** | **OR (95% CI)** | **P value** |
| --- | --- | --- |
| **SPP1 (per SD increase)** | 1.46 (1.28–1.66) | <0.001 |
| Quartile 1 (lowest) | Reference | — |
| Quartile 2 | 1.32 (0.88–1.97) | 0.183 |
| Quartile 3 | 1.89 (1.25–2.85) | 0.003 |
| Quartile 4 (highest) | 2.74 (1.78–4.23) | <0.001 |

**B. Cox proportional hazards analyses for 6-month major adverse cardiovascular events (MACE)**

| **Exposure** | **HR (95% CI)** | **P value** |
| --- | --- | --- |
| **SPP1 (per SD increase)** | 1.41 (1.16–1.71) | 0.001 |
| Quartile 1 (lowest) | Reference | — |
| Quartile 2 | 1.21 (0.72–2.05) | 0.471 |
| Quartile 3 | 1.78 (1.05–3.01) | 0.032 |
| Quartile 4 (highest) | 2.36 (1.38–4.04) | 0.002 |

Serum SPP1 was standardized to a z score, and effect estimates are reported per standard deviation (SD) increase. Quartiles were defined according to the distribution of serum SPP1 levels in the study population, with Quartile 1 serving as the reference category.

Logistic regression models for imaging-defined plaque vulnerability were adjusted as in the primary multivariable model (age, sex, LDL-C, and IL-6). Cox proportional hazards models for 6-month MACE were adjusted as in the primary multivariable model (age, sex, hypertension, LDL-C, and IL-6).

Odds ratios (ORs) and hazard ratios (HRs) are reported with 95% confidence intervals. P values are two-sided and considered statistically significant at *P* < 0.05.

**Supplementary Table S4. Sensitivity analyses for the associations of serum SPP1 with plaque vulnerability and 6-month MACE after additional adjustment for renal function (eGFR)**

**A. Logistic regression analyses for imaging-defined plaque vulnerability**

| **Model** | **Predictor** | **OR (95% CI)** | **P value** |
| --- | --- | --- | --- |
| Primary model* | SPP1 | 1.08 (1.05–1.11) | <0.001 |
| Primary model + eGFR | SPP1 | 1.07 (1.04–1.10) | <0.001 |
| Primary model + eGFR | eGFR | 0.99 (0.97–1.01) | 0.318 |

**B. Cox proportional hazards regression analyses for 6-month MACE**

| **Model** | **Predictor** | **HR (95% CI)** | **P value** |
| --- | --- | --- | --- |
| Primary model† | SPP1 | 1.35 (1.11–1.64) | 0.002 |
| Primary model + eGFR | SPP1 | 1.33 (1.09–1.62) | 0.004 |
| Primary model + eGFR | eGFR | 0.98 (0.95–1.01) | 0.176 |

* Primary logistic regression model adjusted for age, sex, LDL-C, and IL-6.
† Primary Cox proportional hazards model adjusted for age, sex, hypertension, LDL-C, and IL-6.

Renal function was assessed using baseline estimated glomerular filtration rate (eGFR). Sensitivity analyses were performed by additionally adjusting for eGFR to evaluate potential confounding by renal function. Effect estimates for serum SPP1 remained directionally consistent and of similar magnitude after inclusion of eGFR in both logistic and Cox regression models.

All odds ratios (ORs) and hazard ratios (HRs) are reported per unit increase in serum SPP1. P values are two-sided, with P < 0.05 indicating statistical significance.

**Supplementary Table S5. Pairwise comparisons of AUCs for inflammatory biomarkers using DeLong’s test**

| **Comparison** | **AUC (Biomarker 1)** | **AUC (Biomarker 2)** | **ΔAUC** | **P value (DeLong)** |
| --- | --- | --- | --- | --- |
| SPP1 vs IL-6 | 0.76 | 0.64 | 0.12 | 0.003 |
| SPP1 vs MMP-9 | 0.76 | 0.66 | 0.10 | 0.008 |
| SPP1 vs hsCRP | 0.76 | 0.58 | 0.18 | <0.001 |
| IL-6 vs MMP-9 | 0.64 | 0.66 | −0.02 | 0.412 |
| IL-6 vs hsCRP | 0.64 | 0.58 | 0.06 | 0.094 |
| MMP-9 vs hsCRP | 0.66 | 0.58 | 0.08 | 0.031 |

Receiver operating characteristic (ROC) curve analyses were performed to evaluate the discriminatory performance of inflammatory biomarkers for imaging-defined plaque vulnerability. Pairwise comparisons of areas under the ROC curves (AUCs) were conducted using DeLong’s test for correlated ROC curves. ΔAUC represents the difference in AUC between the two biomarkers listed in each comparison.

All P values are two-sided, with P < 0.05 indicating statistical significance.

**Supplementary Table S6. Subgroup analysis for plaque rupture (adjusted OR per 1 SD ↑ in SPP1)**

| **Subgroup** | **n** | **Events, %** | **Adjusted OR (95% CI)** | **P_interaction** |
| --- | --- | --- | --- | --- |
| Overall | 300 | 50.0 | 1.62 (1.34–1.96) | — |
| Sex – Male | 202 | 47.0 | 1.64 (1.29–2.09) | 0.74 |
| Sex – Female | 98 | 56.1 | 1.59 (1.17–2.15) |  |
| Age <65 | 160 | 42.5 | 1.68 (1.28–2.22) | 0.52 |
| Age ≥65 | 140 | 58.6 | 1.55 (1.20–2.00) |  |
| Diabetes – No | 211 | 51.7 | 1.66 (1.31–2.10) | 0.41 |
| Diabetes – Yes | 89 | 46.1 | 1.53 (1.09–2.16) |  |
| Hypertension – No | 144 | 49.3 | 1.67 (1.24–2.25) | 0.81 |
| Hypertension – Yes | 156 | 50.6 | 1.58 (1.23–2.04) |  |
| LDL <3.0 mmol/L | 170 | 41.2 | 1.54 (1.18–2.00) | 0.36 |
| LDL ≥3.0 mmol/L | 130 | 61.5 | 1.71 (1.31–2.23) |  |
| Statin – No | 135 | 48.1 | 1.56 (1.19–2.05) | 0.69 |
| Statin – Yes | 165 | 51.5 | 1.68 (1.31–2.17) |  |
| Smoking – No | 181 | 51.9 | 1.60 (1.25–2.04) | 0.62 |
| Smoking – Yes | 119 | 47.1 | 1.63 (1.22–2.17) |  |
| BMI <25 kg/m² | 140 | 42.9 | 1.58 (1.21–2.06) | 0.78 |
| BMI ≥25 kg/m² | 160 | 56.3 | 1.64 (1.28–2.11) |  |

**Supplementary Table S7. Subgroup analysis for 6-month MACE (adjusted HR per 1 SD ↑ in SPP1)**

| **Subgroup** | **n** | **MACE, %** | **Adjusted HR (95% CI)** | **P_interaction** |
| --- | --- | --- | --- | --- |
| Overall | 300 | 18.0 | 1.44 (1.18–1.76) | — |
| Sex – Male | 202 | 16.3 | 1.45 (1.12–1.89) | 0.86 |
| Sex – Female | 98 | 21.4 | 1.42 (1.05–1.92) |  |
| Age <65 | 160 | 12.5 | 1.47 (1.08–2.00) | 0.77 |
| Age ≥65 | 140 | 24.3 | 1.41 (1.09–1.83) |  |
| Diabetes – No | 211 | 16.6 | 1.45 (1.14–1.85) | 0.93 |
| Diabetes – Yes | 89 | 21.3 | 1.43 (1.03–1.98) |  |
| Hypertension – No | 144 | 15.3 | 1.46 (1.07–1.99) | 0.89 |
| Hypertension – Yes | 156 | 20.5 | 1.42 (1.11–1.82) |  |
| LDL <3.0 mmol/L | 170 | 13.5 | 1.39 (1.04–1.85) | 0.58 |
| LDL ≥3.0 mmol/L | 130 | 22.3 | 1.49 (1.12–1.99) |  |
| Statin – No | 135 | 18.5 | 1.40 (1.05–1.86) | 0.68 |
| Statin – Yes | 165 | 17.6 | 1.47 (1.12–1.93) |  |
| Smoking – No | 181 | 18.2 | 1.46 (1.12–1.90) | 0.83 |
| Smoking – Yes | 119 | 17.6 | 1.41 (1.05–1.90) |  |
| BMI <25 kg/m² | 140 | 14.3 | 1.38 (1.03–1.85) | 0.49 |
| BMI ≥25 kg/m² | 160 | 21.3 | 1.48 (1.13–1.94) |  |

**Supplementary Table S8. Sensitivity analyses for plaque rupture (Logistic)**

| **Specification** | **Adjusted OR (95% CI)** | **P** |
| --- | --- | --- |
| Per 1 SD (primary model) | 1.62 (1.34–1.96) | <0.001 |
| log(SPP1) | 1.58 (1.31–1.91) | <0.001 |
| SPP1 ≥0.48 vs <0.48 | 2.46 (1.64–3.70) | <0.001 |
| SPP1 ≥median vs <median | 2.12 (1.47–3.06) | <0.001 |
| + MMP-9 & IL-6 | 1.38 (1.10–1.74) | 0.006 |
| Trim top/bottom 1% | 1.59 (1.31–1.93) | <0.001 |
| IPTW (stabilized) | 1.57 (1.26–1.96) | <0.001 |
| Multiple imputation (m=20) | 1.61 (1.33–1.95) | <0.001 |

**Supplementary Table S9. Sensitivity analyses for 6-month MACE (Cox)**

| **Specification** | **Adjusted HR (95% CI)** | **P** | **PH-test (Schoenfeld P)** |
| --- | --- | --- | --- |
| Per 1 SD (primary model) | 1.44 (1.18–1.76) | <0.001 | 0.64 |
| log(SPP1) | 1.39 (1.14–1.71) | 0.002 | 0.58 |
| SPP1 ≥0.48 vs <0.48 | 1.88 (1.24–2.84) | 0.003 | 0.71 |
| Alternative MACE definition | 1.37 (1.09–1.72) | 0.006 | 0.52 |
| Restricted to statin users | 1.43 (1.12–1.83) | 0.004 | 0.69 |
| IPTW (stabilized) | 1.42 (1.14–1.77) | 0.001 | 0.66 |
| Multiple imputation (m=20) | 1.43 (1.17–1.76) | 0.001 | 0.61 |
